# Synthesis of CoFe_2_O_4_ magnetic nanoparticles for application in photocatalytic removal of azithromycin from wastewater

**DOI:** 10.1038/s41598-022-21231-2

**Published:** 2022-11-10

**Authors:** Ali Modabberasl, Tahereh Pirhoushyaran, Seyyed Hamid Esmaeili-Faraj

**Affiliations:** 1 Department of Physics, College of Sciences, Yasouj University, Yasouj, 7591874831 Iran; 2 Department of Chemical Engineering, Dezful Branch, Islamic Azad University, Dezful, Iran; 3 Faculty of Material and Chemical Engineering, Shahrood University of Technology, Shahrood, 3619995161 Iran

**Keywords:** Chemical engineering, Nanoscience and technology

## Abstract

Azithromycin is one of the most widely used antibiotics in medicine prescribed for various infectious diseases such as COVID-19. A significant amount of this drug is always disposed of in hospital effluents. In this study, the removal of azithromycin using Cobalt-Ferrite magnetic nanoparticles (MNP) is investigated in the presence of UV light. For this purpose, magnetic nanoparticles are synthesized and added to the test samples as a catalyst in specific proportions. To determine the structural and morphological properties of nanoparticles, characterization tests including scanning electron microscopy (SEM), Fourier transform infrared spectroscopy (FTIR), X-ray diffraction (XRD), vibrating-sample magnetometer (VSM), and Energy-dispersive X-ray spectroscopy (EDX) are performed. 27 runs have been implemented based on the design of experiments using the Box-Behnken Design (BBD) method. Parameters are the initial concentration of azithromycin (20–60 mg/L), contact time (30–90 min), pH (6–10), and the dose of magnetic nanoparticles (20–60 mg/L). The obtained model interprets test results with high accuracy (R^2^ = 0.9531). Also, optimization results by the software show that the contact time of 90 min, MNP dosage of 60 mg/L, pH value of 6.67, and azithromycin initial concentration of 20 mg/L leads to the highest removal efficiency of 89.71%. These numbers are in the range of other studies in this regard.

## Introduction

Drugs are one of the most important sources of water pollution in the world. Statistics show that 90% of them enter the municipal wastewater^1,2^. About 15% of the drugs contain antibiotics, which is a significant source of water contaminants since around 30–90% of the used antibiotics such as azithromycin (one of the most widely used antibiotics in the treatment of infections) are discharged into the sewage without any changes^3,4^. For degradation of these pollutants, different methods have been applied, i.e., filtration, adsorption, electrochemical techniques, ozonation, advanced oxidation, and photocatalytic processes^5–15^.

Azithromycin with the chemical formula of C_38_H_72_N_2_O_12_ and molecular weight of 748 g/mol is a powerful macrolide antibiotic used to treat some bacterial infections such as middle ear infection, streptococcal sore throat, pneumonia, and some other gastrointestinal inflammations. Besides, it is widely prescribed for cases of covid-19 and one of the most effective antibiotics to cure severe infections as well^1,16^.

Remediation of azithromycin by a sonochemical process in the presence of zinc oxide nanoparticles led to the efficiency of 98.4% under the optimum pH = 3, temperature = 40 ºC after 15 min^1^. Another research revealed that azithromycin removal was 58%, just under ultraviolet radiation and close to complete removal when it combined with sodium persulfate (UV/Na_2_S_2_O_8_)^17^. The use of UV/H_2_O_2_ along with moving-bed biofilm reactor (MBBR) systems has led to an appropriate result in removing this pollutant. Besides, it was removed up to 78.3% by BWO-GO photocatalysts^18^. Salles et al. reported the adsorption of azithromycin by magnetic nanocrystalline cellulose. The results showed that the maximum adsorption was reached at pH 3.0 using NC‧Fe_3_O_4_ 1:10. They concluded that adsorption efficiency was highly dependent on experimental conditions^19^.

Nowadays, magnetic nanoparticles and especially, iron magnetic nanoparticles have turned into a rather powerful method to remove pollutants from wastewaters and it is because of their high performance achieved by high surface area per volume, high capacitance in adsorption, high disposability, low mass transfer limitation, low toxicity, and ease of the separation in comparison with other catalysts such as CuS^20,21^. In addition, researches show that by modifying the surface of MNPs the performance of treatment can increase especially because of their effect on bacterial agents^22–24^. Mg_0.5_Ni_0.5_Al_x_Fe_2-x_O_4_ MNPs had a performance of 94% for removal of blue 129 dye^25,26^. On the other hand, the influence of MNPs in eliminating antibiotics from wastewater is considerable. Kamranifar et al. investigated the effect of CoFe_2_O_4_@CuS on Penicillin G (PG) degradation and reached an efficiency of 70.7%^27^. Two similar studies used FeNi_3_/SiO_2_/CuS^28,29^ for the treatment of tetracycline and led to a complete removal, while the use of FeNi_3_@SiO_2_( without CuS agent) magnetic nanoparticles caused an efficiency about 87%^29^. The implement of GO@Fe_3_O_4_/ZnO/SnO_2_ nanocomposite caused to the elimination of 90.6% of azithromycin in an aquatic environment^30^.

This work focuses on the degradation of the azithromycin in aqueous solutions by a photocatalytic process in the presence of cobalt ferrite magnetic nanoparticles. Previous studies have demonstrated that they can’t succeed in high removal efficiency for azithromycin, as well as the problems of separating the degradation agent (nano catalyst) from the treated wastewater. In this research, not only a high efficiency is achieved, but also MNPs can be separated easily from the wastewater due to the magnetic properties. This issue is extremely crucial in operational conditions. After characterizing the synthesized nanoparticles, the effect of contact time, initial azithromycin concentration, pH, and nanoparticle content were investigated using the design of experiments. Finally, the results obtained under optimal conditions are compared with other studies.

## Methods and materials

### Materials

In this study, Azithromycin (C38H72N2O12) with a purity of more than 98% (Sigma Aldrich) was prepared by the Daya Exir Company (Tehran, Iran). In addition, Sodium hydroxide (NaOH with a purity of more than 98%, iron (III) nitrate (Fe(NO_3_)_3_.9H_2_O), Cobalt (II) nitrate (Co(NO_3_)_2_.6H_2_O), Hydrochloric acid (HCl), and ethanol (C_2_H_5_OH) were purchased from Merck Company, (Germany).

### Synthesis of CoFe_2_O_4_ magnetic nanoparticles

For synthesizing CoFe_2_O_4_ magnetic nanoparticles, two components, including Iron (III) nitrate and Cobalt(II) nitrate, are needed. At first, the amount of 0.001 mol of cobalt nitrate and 0.002 mol of iron nitrate are solved in 250 mL deionized water separately, and after that, they are mixed. Then this solution is combined with 20 mL of NaOH (0.1 Molar), and the mixture is stirred for 2 h at 80 °C. In this step, cobalt ferrite nanoparticles are formed and the sediments are separated via the magnet. The final step is washing in deionized water and ethanol.

### Apparatus

To identify the synthesized nanoparticles, field emission scanning electron microscopy analysis (FESEM), energy dispersive X-ray analysis (EDS, Sigma 300-HV Zeiss, Germany), X-ray diffraction analysis (XRD, Unisantis XMD300, Germany), Zeta potential analysis (Paar, Austria), and vibrating-sample magnetometer (VSM1100, Weistron, Shanghai, China), Brunauer- Emmett- Teller (BET) analysis (Belsorp miniII, Japan) were carried out.

The system used for the photocatalytic process consists of a pyrex container with dimensions of 20 × 10 × 10 cm. The simulated effluent contains different concentrations of azithromycin with catalytic nanoparticles, which are uniformly dispersed in the effluent at the bottom of the container (with a depth of 3 cm). The end of the reactor is a conduit for expelling the contents and sampling and the output can be returned to the reactor. The cause of liquid circulation in the reactor is mixing in the reactor contents and uniformity of the pollutant concentration in the entire reactor contents. A BQ50-1 J peristaltic pump (Partoshar Company, Iran) has been used to circulate the solution. In this apparatus, a lamp with a length of 20.1 cm and the type of UV-C—18 W (Philips, Poland) is located at the top of the reactor, which can produce a wavelength of 253.7 nm; its lifetime is 8000 h with the radiation intensity of 294–282 W/m^2^ at 1 cm of distance.

The UV lamp is placed on top of the reactor at a distance of 5 cm to the liquid level. The entire reaction chamber is covered by aluminum foil to prevent UV absorption from the environment. The schematic of the reactor set is shown in Fig. S1 in the supplementary section. A high-performance liquid chromatography device (HPLC, Agilent Corp., USA) is used to analyze the azithromycin concentrations in the samples.

### Experiment procedure

The experiments are performed by addition of 650 mL of solution with different concentrations of azithromycin (as a contaminant) and magnetic nanoparticles (as a catalyst) according to the design of the experiments. To achieve stable conditions, the contents of the reactor are first circulated for 5 min without UV light, and after establishing a steady state, the UV lamp turns on. During the reaction period, 5 mL of solution is collected from the sample site and sent for analysis via HPLC at specific times according to the design of the experiments. Before HPLC analysis, nanoparticles are first separated from the liquid phase by a strong magnet placed on the outer surface of the sampling vessel, and then, a clear liquid sample free of nanoparticles is used for testing.

### Design of experiments

Numerous parameters affect the performance of advanced oxidation processes among which, four factors were examined in this study according to the nature of the process and the available facilities. The initial concentration of azithromycin (pollutant) in the inlet effluent is one of the most influential factors. These values are 20, 40, and 60 mg/L. Process time plays an essential role in removal efficiency and so, values ​​of 30, 75, and 120 min were considered to examine the concentration of contaminants. In some references, longer time values have been selected. Still according to the initial performed tests, it was observed that at times above 100 min, the pollutant concentration changes very slowly and the slope of changes is higher at times below 100 min. Since, the pH of the solution is effective on the stability of the combination of azithromycin and nanofluids^20^, three pH levels of 6, 8, and 10 were studied. To evaluate the amount of catalyst, concentrations of 20, 40, and 60 mg/L were used. Table 1 depicts the list of studied parameters and their levels (coded as − 1, 0, and + 1). The response parameter is the removal efficiency (RE) that is obtained by Eq. (1) using final concentration of the pollutant. The experiments are according to the Box-Behnken (BBD) method which is one of the types of response surface methodology (RSM).

**Table 1:** List of studied parameters and evaluated levels.

Parameter	Unit	Levels		
		− 1	0	1
A: Az initial concentration (${\mathrm{C}}_{\mathrm{Az},0}$)	mg/L	20	40	60
B: Contact time (t)	min	30	60	90
C: pH value (pH)	–	6	8	10
D: MNP dosage (${C}_{cat}$)	mg/L	20	40	60

1 $$RE=\frac{Az\,\, initial \,\,concentration-Az \,\,final \,\,concentration}{Az \,\,initial \,\,concentration}\times 100$$

## Results and discussion

### Characterization of magnetic nanoparticles

**Figure 1:**
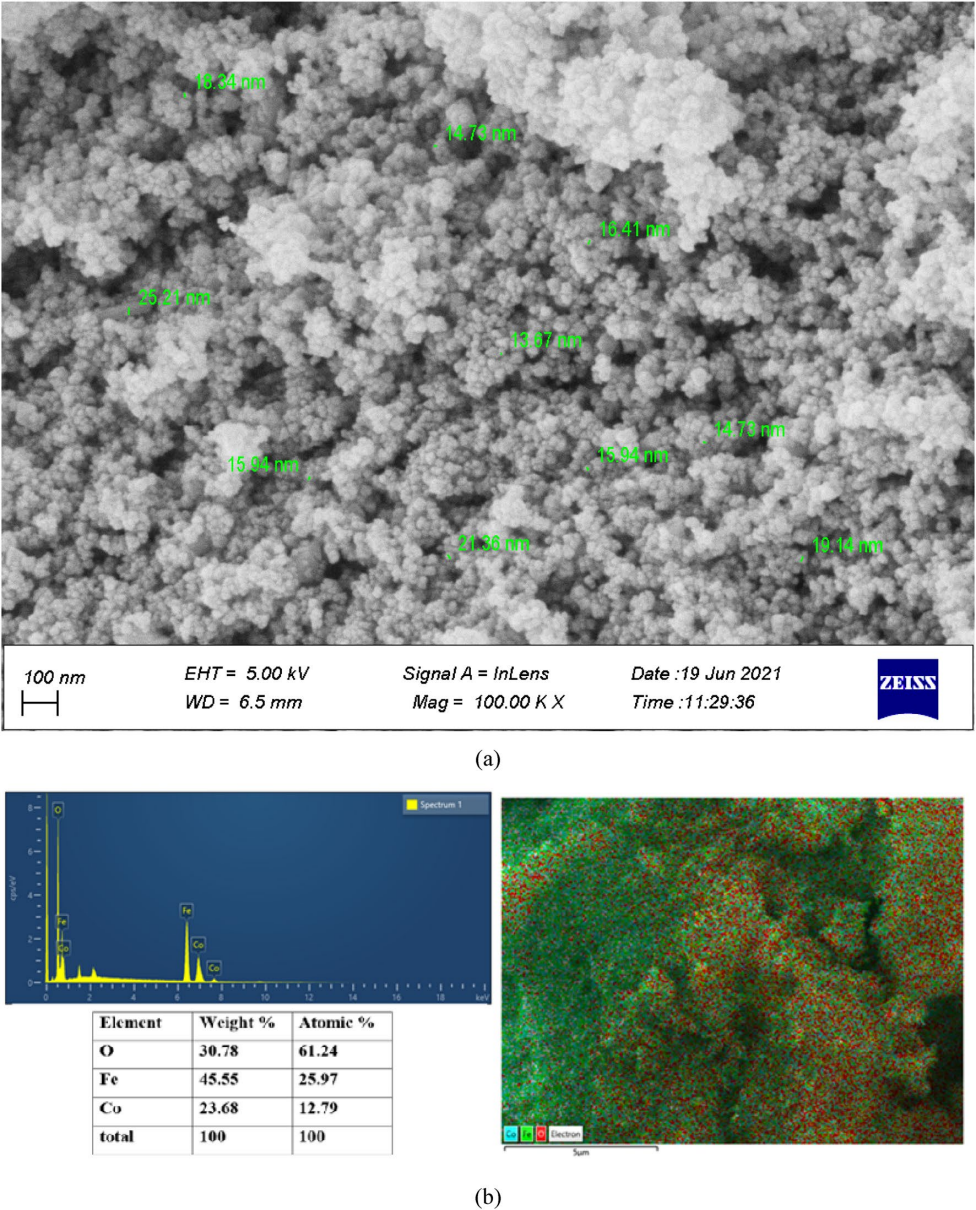
The results of (**a**) FESEM analysis; (**b**) EDS analysis, for CoFe_2_O_4_ MNP.

Figure 1a shows the FE-SEM images of CoFe_2_O_4_ nanoparticles. The spherical structure of nanoparticles with particles size of less than 25 nm is evident in the figure. The results of the EDS analysis are presented in Fig. 1b. According to this analysis, the presence of oxygen, iron, and cobalt atoms in the structure of nanoparticles is 61.24, 25.97, and 12.79 percent, respectively. The weight fractions are 30.78, 45.55, and 23.68, respectively. Also, the figure shows that the distribution of these atoms at the surface is almost uniform.

**Figure 2:**
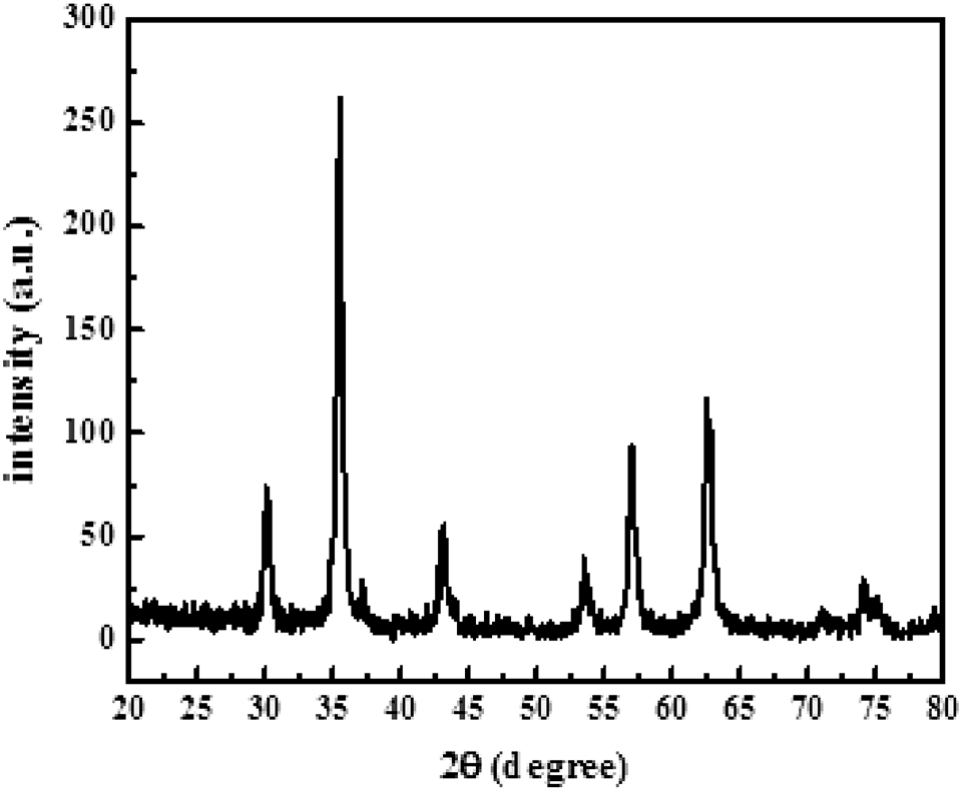
Results of XRD analysis for CoFe_2_O_4_ MNP.

The X-ray diffraction (XRD) pattern of the CoFe_2_O_4_ nanoparticles is shown in Fig. 2. From the patterns in the range of 2θ = 20°–80°, the reflecting planes (220), (311), (222), (400), (422), (511), (440), and (442) confirm the formation of single phase cubic spinel structure of CoFe_2_O_4_ ferrite with the Fd-3 m space group (According to the standard CoFe_2_O_4_ powder diffraction JCPDS card no. 22-1086). No sign of a secondary or impurity phase was detected in the pattern of the prepared sample.

**Figure 3:**
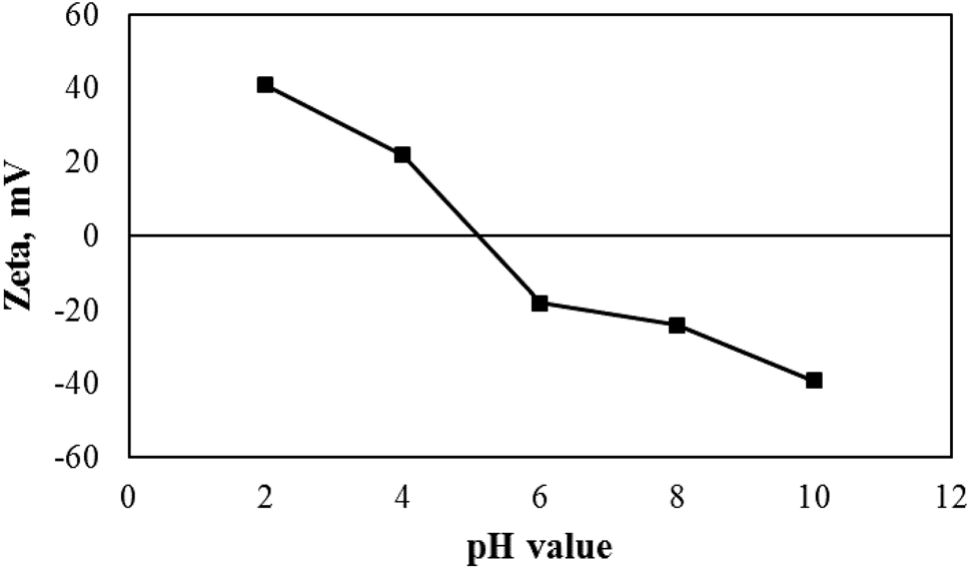
Results of Zeta potential measurement versus pH values for CoFe_2_O_4_ MNP in water.

Figure 3 shows the results of zeta potential analysis in terms of pH values. This test is an indicator of the number of surface charges of nanoparticles. There is relative stability in the nanofluid at zeta potential values above 20 or below -20. The nanofluid will be quite stable at values of about 40 and -40. Accordingly, for pH values less than 4 to more than 6, we will see relatively good stability of nanoparticles in aqueous solutions. Therefore, the pH values equal to 6, 8, and 10 was considered for experimental tests.

**Figure 4:**
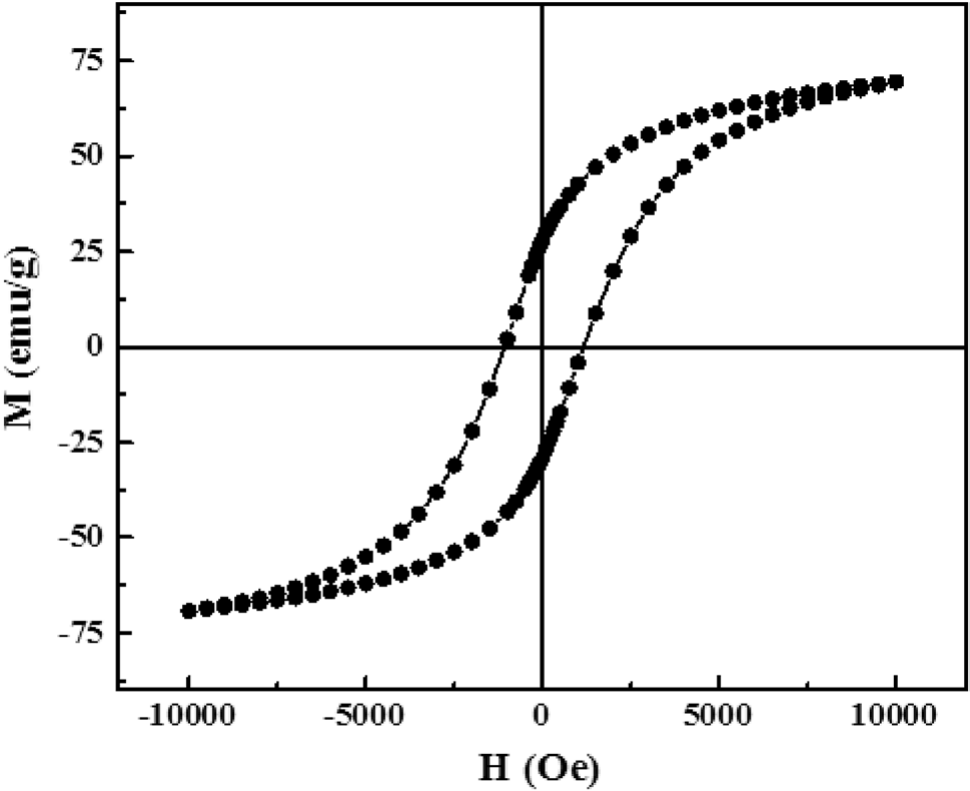
The typical recorded hysteresis loop of the CoFe_2_O_4_ magnetic nanoparticles.

Magnetic features of the CoFe_2_O_4_ magnetic nanoparticles were measured at the temperature of 300 K with a magnetic field up to $\pm$ 10 kOe. Figure 4 displays the magnetic sample's typical recorded hysteresis loop. The cobalt ferrite shows a ferromagnetic behavior with the saturation magnetization (M_S_) of about 69 emu/g. This result (M_S_ value) is similar to those reported by other studies^31,32^ for CoFe_2_O_4_ magnetic nanoparticles. Mosleh et al.^33^ reported the maximum M_S_ value of 46 emu/g for BaFe_12_O_19_ magnetic nanoparticles. Lakhani et al.^34^ reported the M_S_ value of about 58 emu/g for CuFe_2_O_4_ magnetic ferrite. Rhein et al.^35^ also reported a better M_S_ value (about 71 emu/g) for SrFe_12_O_19_ magnetic ferrite. Another report by Borhan et al.^36^ indicates the M_S_ value of about 3 emu/g for ZnFe_2_O_4_ ferrite.

One of the indicators to identify nanomaterials is their specific surface area. Since nanoparticles are very small particles, their surface-to-volume ratio is very high. Therefore nanoparticles also exhibit a relatively high surface area per unit mass compared to micrometer-sized particles^37^. The specific surface area of porous, non-porous materials and also nanoparticle materials can excellently be measured and quantified by the BET analysis using physical gas adsorption of nitrogen gas at an analysis temperature of 77 K. The specific surface area obtained by this analysis is called BET specific surface area (*a*_*s,BET*_) and expressed in m^2^/g. A summary of the results of the BET test is presented in Table 2. According to that, the specific surface area for nanoparticles is 56.773 m^2^/g. This value is expected for non-porous nanoparticles, so the particles are on a nanoscale.

**Table 2:** Summary of results of BET test for the CoFe_2_O_4_ MNPs.

Parameter	Value	Unit
Volume of the adsorbed gas: *V*_*m*_	13.044	cm^3^(STP)/g
Specific surface area: *a*_*s,BET*_	56.773	m^2^/g
Total pore volume(*p/p*_*0*_ = 0.990)	0.1417	cm^3^/g
Mean pore diameter	9.9836	nm

### Results of BBD methodology

The experimental conditions for effective parameters, including the initial azithromycin concentration, contact time, pH value, and MNP dosage, as well as the removal efficiency of azithromycin as the response of the BBD method, are presented in Table 3.

**Table 3:** Experiment conditions using BBD method.

Run	A: Az initial concentration, mg/L	B: Contact time, min	C: pH value	D: MNP dosage, mg/L	Final Az Concentration, mg/L	RE, %
1	40	90	6	40	24.3	0.73
2	40	30	6	40	77.39	0.14
3	20	60	8	60	3.34	0.89
4	40	60	8	40	43.19	0.52
5	20	60	8	20	11.08	0.63
6	20	60	6	40	18.31	0.39
7	60	90	8	40	39	0.74
8	60	60	6	40	102	0.32
9	40	30	8	60	27.91	0.69
10	20	30	8	40	13.52	0.55
11	60	60	8	20	94.46	0.37
12	60	30	8	40	106.48	0.29
13	40	60	8	40	37.84	0.58
14	40	90	8	60	5.45	0.94
15	40	60	10	20	68.44	0.24
16	40	60	8	40	35.08	0.61
17	40	90	8	20	22.54	0.75
18	40	30	10	40	62.11	0.31
19	60	60	8	60	58.53	0.61
20	20	60	10	40	11.07	0.63
21	60	60	10	40	108.02	0.28
22	40	60	10	60	53.09	0.41
23	40	60	6	20	73.76	0.18
24	40	90	10	40	63.89	0.29
25	40	60	6	60	52.22	0.42
26	40	30	8	20	66.57	0.26
27	20	90	8	40	2.73	0.91

The results of ANOVA based on design of experiments are shown in Table 4.

**Table 4:** Results of ANOVA by Design Expert software.

Source	Mean square	F-value	p-value
Model	0.0950	17.43	< 0.0001
A-Az initial concentration	0.0369	6.76	0.0232
B-time	0.1680	30.84	0.0001
C-pH	0.0008	0.1380	0.7167
D-MNP dosage	0.0114	2.09	0.1735
AB	0.0020	0.3717	0.5534
AC	0.0196	3.60	0.0822
AD	0.0001	0.0184	0.8945
BC	0.0930	17.08	0.0014
BD	0.0144	2.64	0.1299
CD	0.0012	0.2249	0.6439
A^2^	0.0096	1.77	0.2083
B^2^	0.0091	1.67	0.2211
C^2^	0.3104	56.98	< 0.0001
D^2^	0.0012	0.2203	0.6472
Lack of fit	0.0061	2.91	0.2825
Std. dev	7.38		
R^2^	0.9531		
Adjusted R^2^	0.8984		
Predicted R^2^	0.7406		

F-value for the model equals 17.43 shows the model is significant. Also, it can be said that there is just a probability of 0.01% for occurring this large amount of F-value because of the noise. On the other hand, whenever the amounts of P-values are less than 0.05, model parameters are significant. So, A, B, BC, and C^2^ are significant. On the contrary, P-values more than 0.1, indicate the model parameters are not significant. F-value is 2.91, and emphasizes that lack of fit is not significant rather than the pure error. The amount of 28.25% in this row for P-values is related to the probability of occurring due to the noise. Since the difference between the predicted R^2^ (0.7406) and the adjusted R^2^ (0.8984) is less than 0.2, so they are in a reasonable agreement together. R-square of 0.9531 denotes a proper fitting of the models on data. So, the model equation is as follows:

2 $$\mathrm{RE}=-421.11458-0.379167\times {\mathrm{C}}_{\mathrm{Az},0}+2.06806\times t+108.35417\times pH+1.29375\times {C}_{cat}+0.003750\times {\mathrm{C}}_{\mathrm{Az},0}\times t-0.175\times {\mathrm{C}}_{\mathrm{Az},0}\times pH-0.00125\times {\mathrm{C}}_{\mathrm{Az},0}\times {C}_{cat} -0.254167\times t\times pH-0.01\times t\times {C}_{cat}-0.04375\times pH\times {C}_{cat}+0.010625\times {C}_{Az,0}^{2}+0.004583\times {t}^{2}-6.03125\times p{H}^{2}+0.00375\times {C}_{cat}^{2}$$

**Figure 5:**
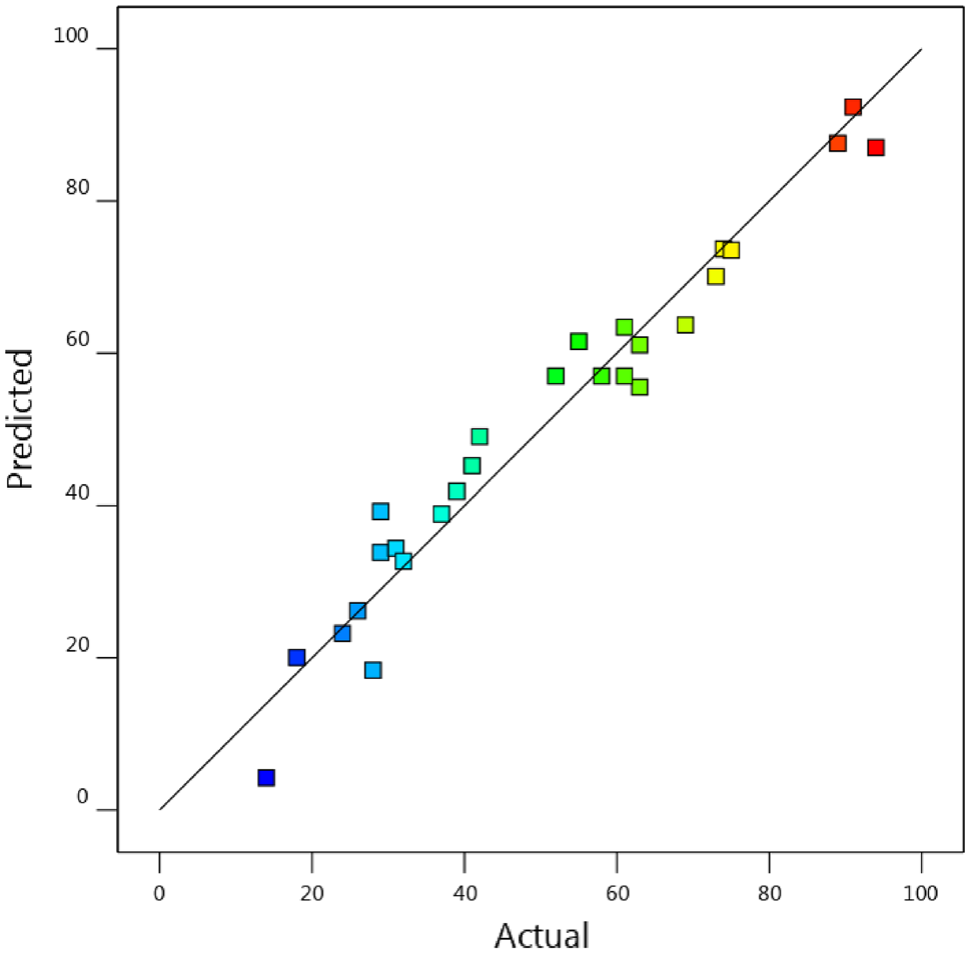
Predicted values of RE vs. actual values obtained by Design Expert.

That ${\mathrm{C}}_{\mathrm{Az},0}$, $t$, $pH$, and ${C}_{cat}$ are the azithromycin initial concentration, contact time, pH value, and MNP dosage respectively. The equation can be a helpful relationship to predict the response for certain levels of each factor in terms of actual factors. Of course, each level should be determined according to the original unit of each factor, as in Table 1. Another point regarding the equation is that it cannot be used for determination of the relative impact of the factors, since these coefficients are scaled to match the units of each term. In Fig. 5, the predicted values of RE versus the actual values from Table 3 have been shown. In Fig. 5, almost all the points correspond to the bisector (y = x), and this is on the ground that, the fit of model on data is appropriate.

### Effects of parameters

**Figure 6:**
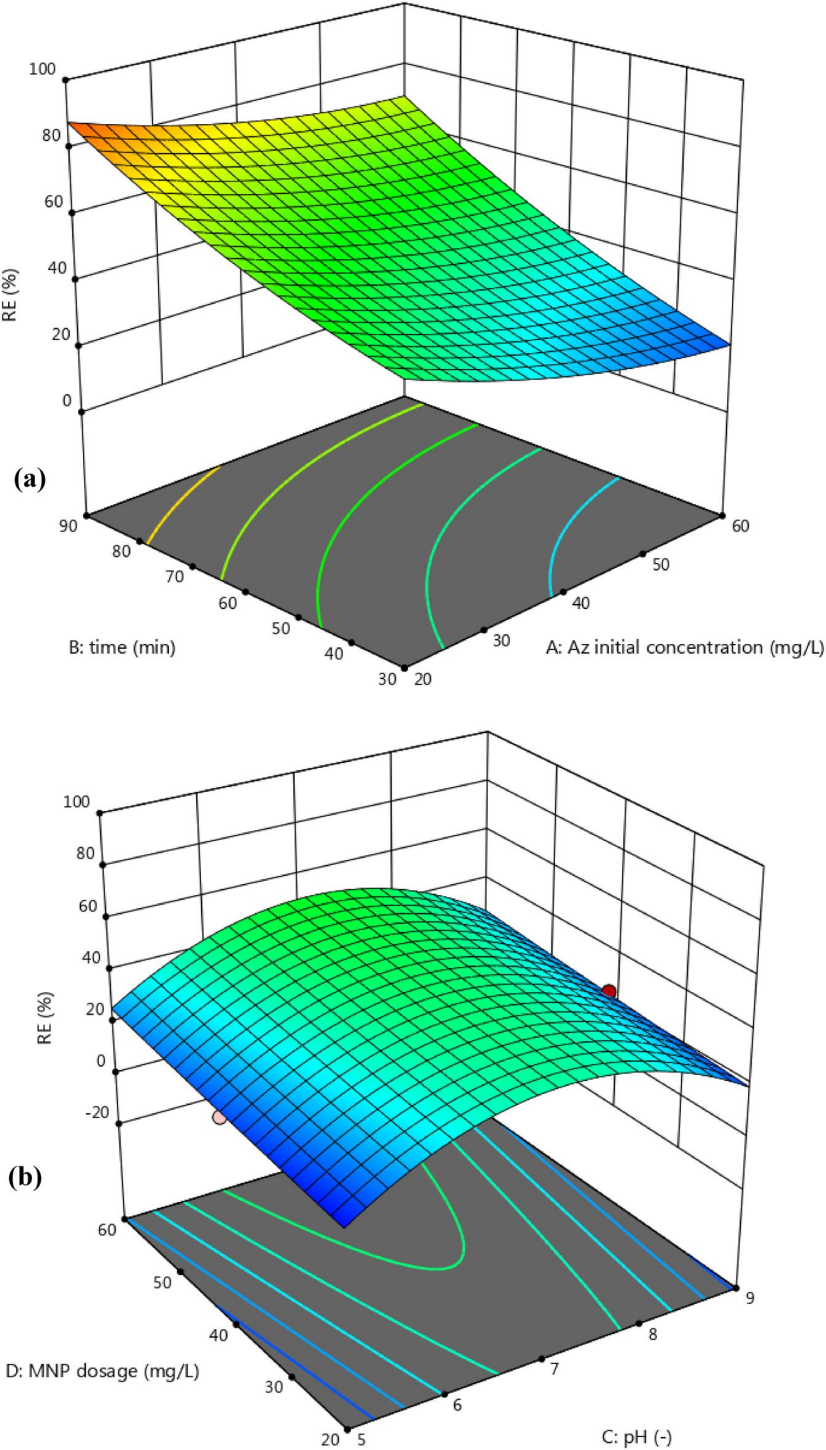
Effect of parameters (**a**) time and Az initial concentration, (**b**) MNP dosage and pH value, on removal efficiency.

The effect of studied factors on the removal efficiency is shown in Fig. 6a,b. According to these graphs, it is concluded that the removal efficiency decreases uniformly with increasing the initial concentration of the contaminant. While with increasing contact time and catalyst concentration, the removal efficiency will increase. The rate of change of the removal efficiency will be almost the same as the changes in the initial concentration and contact time, whereas in contrast, the removal efficiency will increase with a less intensity as the amount of catalyst increases. The effect of pH on the RE is in a way that increasing the pH firstly causes to increase in RE, and then decrease. So, there is a maximum point for the removal efficiency, and that is the middle of pH range. Figure S2 (in the supplementary) presented the other interaction parameters and their effects on RE.

Optimal operating values can be achieved with Eq. 3 to have the maximum removal efficiency. In obtained results from the software, it can be said that the highest level of contact time (90 min), and catalyst concentration (60 mg/L), intermediate pH values (6.67) and low pollutant values (20 mg/L) lead to the highest removal efficiency of 89.71%.

**Figure 7:**
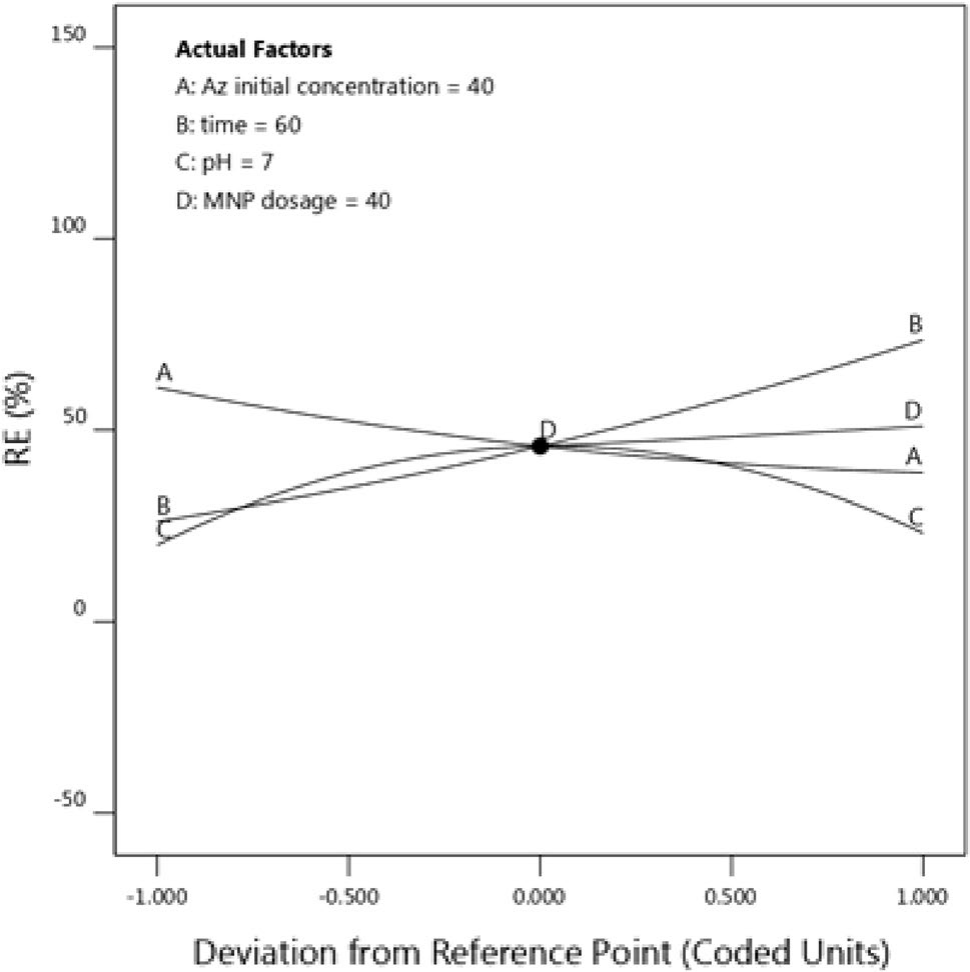
Perturbation curve for removal efficiency versus coded parameters.

The effect of investigated parameters is presented in the perturbation diagram (Fig. 7). The curves of this graph are obtained by use of Eq. (2), and each curve is obtained by the different values of the considered parameter in Eq. (2) and the central values of other parameters. It can be seen that the removal efficiency increases with time because the contact time between the catalyst and the pollutant has increased, and therefore, the removal efficiency will increase.

As the initial concentration of azithromycin increases, the removal efficiency decreases due to the increase in the loading rate. When nanoparticle concentration increases, the removal efficiency will also increase due to the promotion of the active surface for the reaction. Removal efficiency is maximum at neutral pH values and decreases as moving away from the neutral range. At pH values of 5, the value of zeta potential is close to zero, and therefore, because of the low stability of the nanofluid, the RE decreases. At pH values close to 9, due to the alkalinity of the reaction media, *OH*^*-*^ anions compete with the catalysts to react with the pollutant, and the removal efficiency of these ions is slightly lower than that of the nano-catalyst.

To determine whether azithromycin is degraded or adsorbed on the surface of the catalyst, after two selected runs (Run 3 & 19), two catalyst samples were collected at the end of the process and washed with 5 cc of deionized water, and the deionized water sample was collected for the HPLC test. The first sample contained only 2 ppm of azithromycin and the second sample contained 2.4 ppm. HPLC results for these two samples show that a tiny amount of azithromycin was absorbed on the catalyst’s surface, and so, a significant part of the missing azithromycin was degraded by the catalyst.

Table 5 shows the removal of drug compounds by photocatalytic process under optimal conditions. As shown in Table 4, different catalysts have been used to remove a mix variety of drugs. However, utilizing CoFe_2_O_4_ as a MNP catalyst has advantages such as high efficiency, low MNP dosage, medium reaction time, pH in the range of 6–7, and easy operation.

**Table 5:** The removal of drug compounds by photocatalytic methods under optimal conditions using various catalysts.

Drug	NPs types	Drug concentration	NP dose, mg/mL	Contact time	pH	RE%	References
Azithromycin	CoFe_2_O_4_	20 mg/L	0.06	90 min	6.67	89.7	This work
Penicillin G	CoFe_2_O_4_@CuS	10 mg/L	0.2	120 min	5	70.7	^27^
tetracycline	FeNi_3_/SiO_2_/CuS	10 mg/L	0.005	200 min	9	100	^38^
penicillin G	β-Lactamase/Fe_3_O_4_	1.1 mM	0.5	10 min	7	98	^21^
tetracycline	FeNi_3_@SiO_2_	10 mg/L	0.1	180 min	7	87	^29^
Meloxicam	gallic acid-	100 mg/L	5	480 min	9	89.1	^39^
Humic acid	FeNi_3_@SiO_2_@TiO_2_	10 mg/L	0.01	30 min	3	100	^40^
Azithromycin	ZnO_2_	20 mg/L	–	15 min	3	98.4	^1^
Azithromycin	GO@Fe_3_O_4_/ZnO/SnO_2_	30 mg/L	1	120 min	3	90	^30^

## Conclusion

In this study, the removal of azithromycin contaminant, a potent antibiotic, was investigated by a photocatalytic process. Cobalt ferrite (CoFe_2_O_4_) magnetic nanoparticles have been used as catalysts along with UV rays. The parameters studied are the initial concentration of azithromycin (20–60 mg/L), contact time (30–90 min), pH (6–8), and the dose of magnetic nanoparticles (20–60 mg/L). The results of the experiments show that with optimal operating conditions (*t* = 90 min, ${C}_{cat}=$ 60 mg/L, *pH* = 6.67, and ${C}_{Az,0}=$ 20 mg/L), removal efficiency was up to 89.7%. According to the comparison between the results of this work and other researches, it can be concluded that cobalt-based nanoparticles have an outstanding ability to remove antibiotics, such as azithromycin. Magnetic properties of nano-catalysts improve their separation process from treated wastewater, and it is an advantage over other non-magnetic nanoparticles.

## Supplementary Information

Supplementary Information.

## Data Availability

All data generated or analyzed during this study are available on reasonable request from the corresponding author.
