# Supplementary material for: Synthesis of CoFe2O4 magnetic nanoparticles for application in photocatalytic removal of azithromycin from wastewater
Source: Sci Rep. 2022 Nov 10;12:19171. doi: 10.1038/s41598-022-21231-2 (PMC9647251; doi:10.1038/s41598-022-21231-2)
Supplement: Supplementary file 1 — Supplementary Information. [file 41598_2022_21231_MOESM1_ESM.docx]

**Synthesis of CoFe_2_O_4_ Magnetic Nanoparticles for Application in Photocatalytic Removal of Azithromycin from Wastewater**

Ali Modabberasl ^1^, Tahereh Pirhoushyaran ^2^, Seyyed Hamid Esmaeili-Faraj ^3*^

^1^ Department of Physics, College of Sciences, Yasouj University, Yasouj, 7591874831, Iran

^2^ Department of Chemical Engineering, Dezful Branch, Islamic Azad University, Dezful, Iran

^3^ Faculty of Material and Chemical Engineering, Shahrood University of Technology, Shahrood, 3619995161, Iran.

*Corresponding author: [h.esmaeili@shahroodut.ac.ir](mailto:h.esmaeili@shahroodut.ac.ir)

**Supplementary Section:**

Set-up of the experimental test are presented in figure S1.


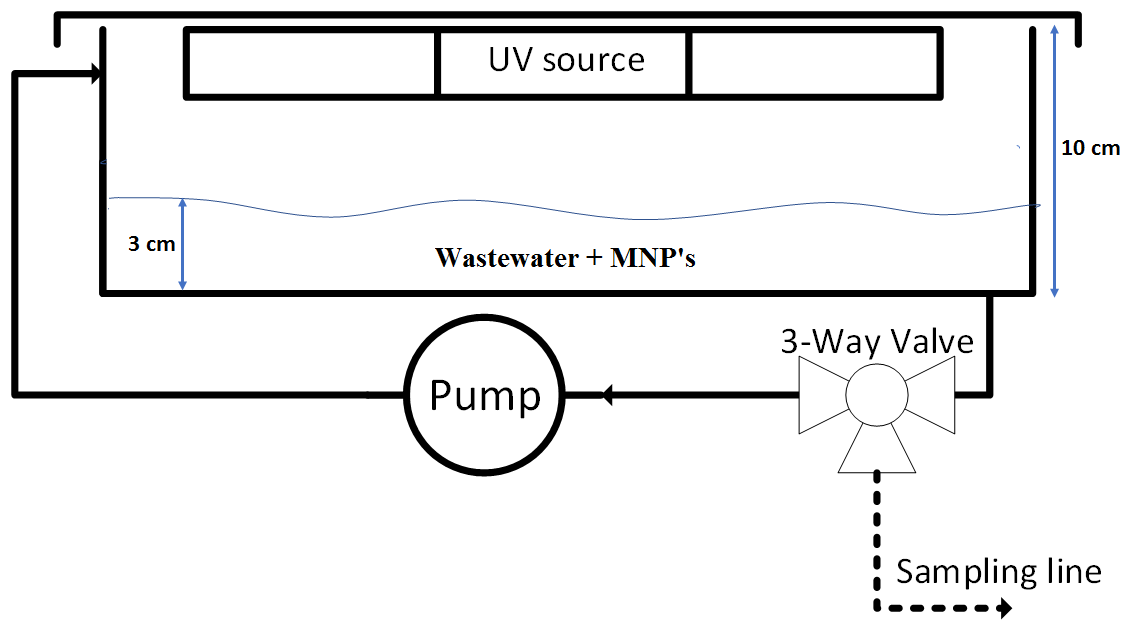


Fig. S1: Schematic of the set-up for photocatalytic process


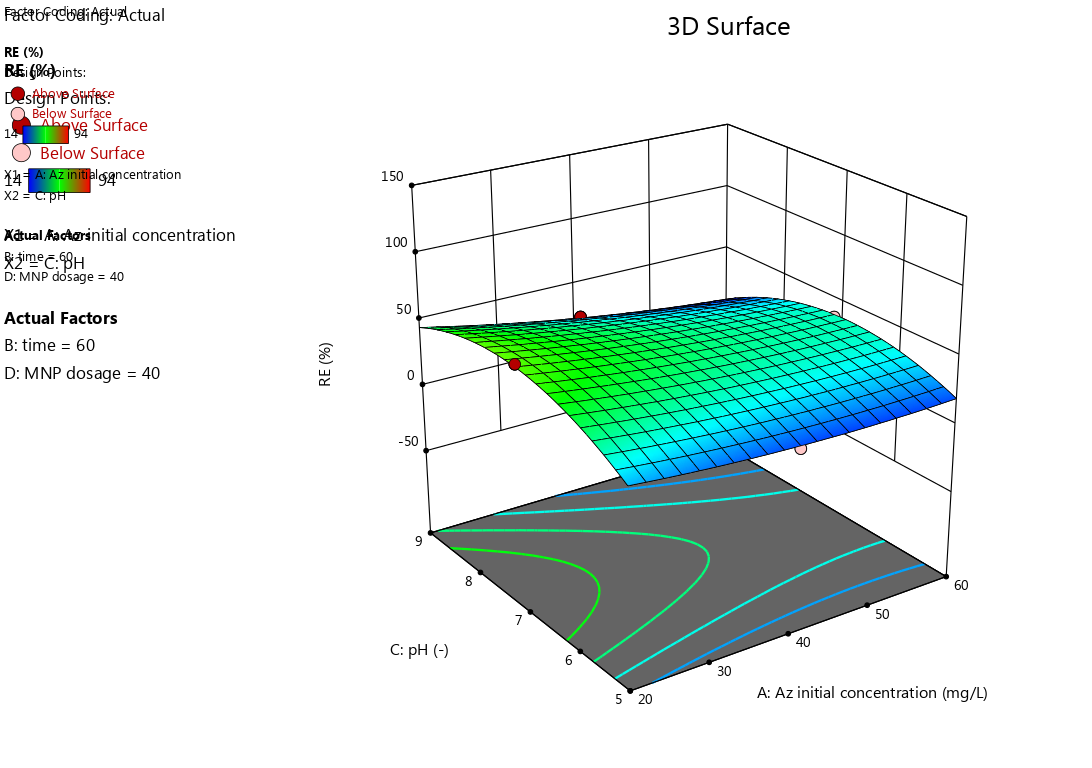


**(a)**


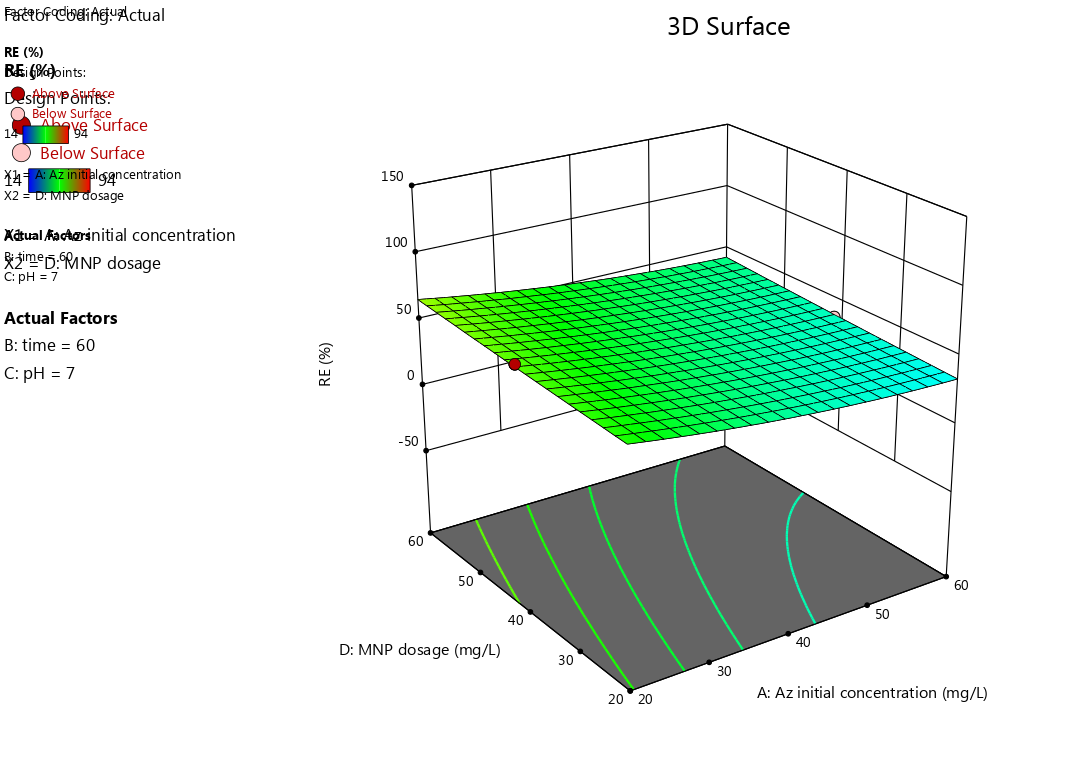

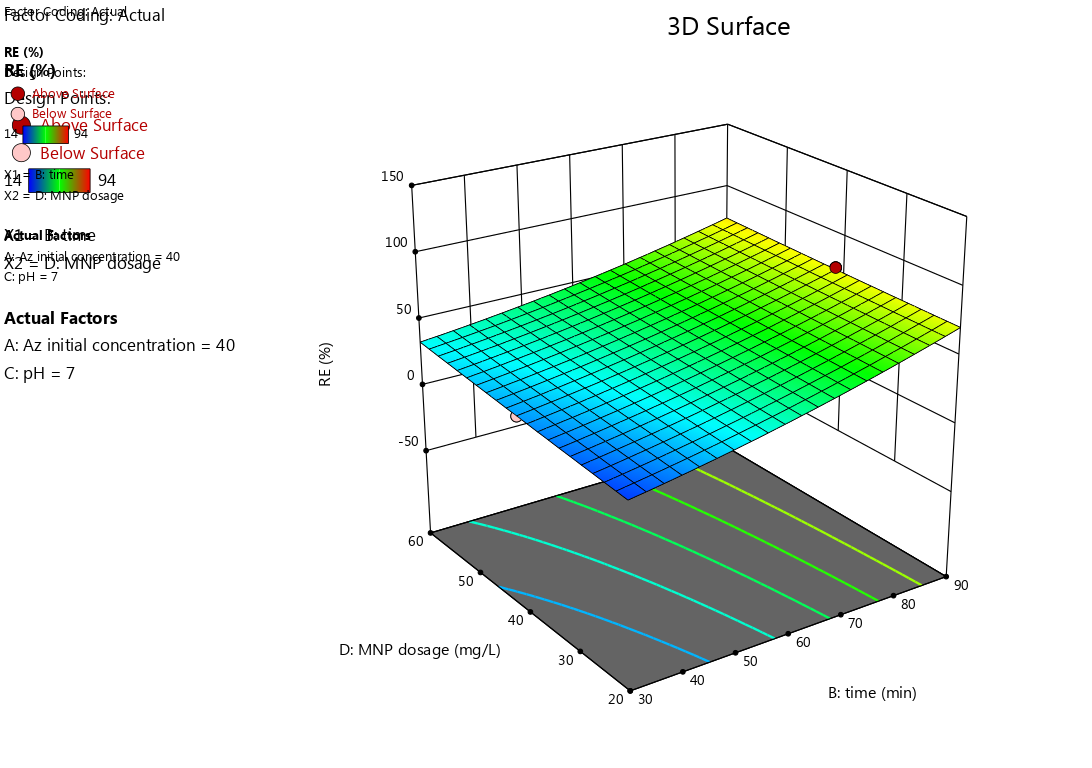


**(c)**

**(b)**


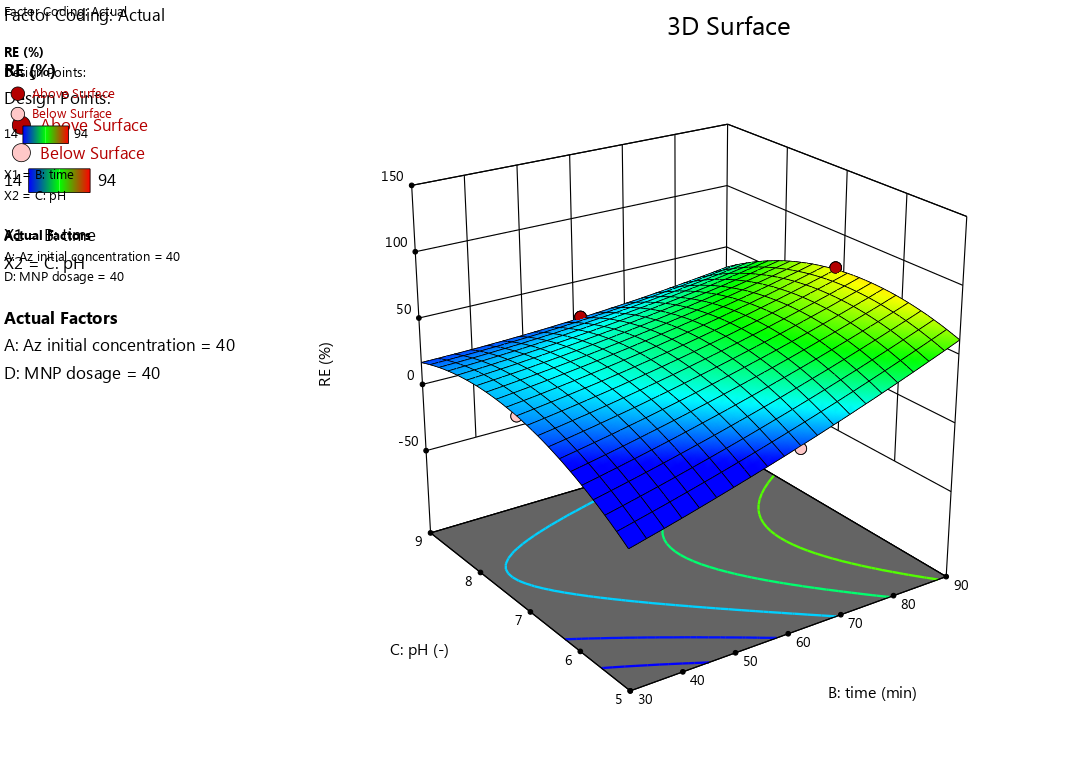


**(d)**

Fig. S2. Effect of parameters (a) pH and Az initial concentration, (b) MNP dosage and Az initial concentration, (c) MNP dosage and time, and (d) pH and time, on removal efficiency
